# James Robinson, Esq.

**Published:** 1862-05

**Authors:** 


JAMES ROBINSON, ESQ.—The proverbial uncertainty of life has, perhaps, seldom been more sadly exemplfied than in the case of the late Mr. Robinson. Our readers have learnt from the columns of the public press the main particulars relating to his lamented death :—that while walking in the garden of his suburban residence, Kenton, near Harrow, a stray branch attracted his attention, and he proceeded to sever it. The instrument (a new pruning knife) cut suddenly through, and, from the force of the stroke, penetrated his left thigh, about two inches above the knee. There was scarcely any hemorrhage at the time; and although Mr. Robinson was at first afraid that the main artery was wounded, after applying a little plaister and binding the limb round with a pocket-handkerchief, his anxiety abated, and he thought little more of it. His wife persuaded him, however, to go to bed, and immediately, without Mr. Robinson's knowledge, sent for medical aid, not judging the wound to be a serious one, but simply for satisfaction's sake. On the arrival of Messrs. Bridgewater from Harrow, Mr. Robinson had lost so much blood that they found him in a state of collapse, from which he never rallied, but gradually became weaker and weaker, and finally sunk from pure exhaustion at 12.30 A.M., on Tuesday morning.

Mr. Robinson has occupied for many years so prominent a position in the profession, both as a practitioner and as a reformer, that a sketch of his career, however brief, will be read with interest. That this should not be more ample in detail, is attributable to the difficuly of obtaining the necessary information in the short space of time at our disposal.

James Robinson was the youngest son of the late Captain Chas. Robinson, R. N., who was for several years one of the Commanders of Greenwich Hospital. He was a native of Hampshire, and was born on the 22d of November, 1813, consequently he was forty-eight years of age at the time of his death. From an early period, energy and activity gave indications of the perseverance and enterprise which were characteristic of maturity. At fourteen years old he was articled to a surgeon and chemist in London, and at the close of his indentures entered himself at Guy's Hospital, and the London University, where he pursued his studies with considerable ardor, and devoting especial attention to the structure and diseases of the teeth. In 1830 he commenced business in Store street, Bedford square, we have been given to understand, as a chemist. His predilections, however, quickly led him to adopt the branch of practice to which he had given so much attention, and his dexterity as a dentist soon became known. By strict attention to the duties of his practice, the latter gradually extended. In 1834 he received the appointment of Surgeon-Dentist to the Metropolitan Hospital, (now the Royal Free Hospital), and from this period dates his remarkable success in his profession. In 1840 he removed to Gower street, Bedford square, and it was here that he first introduced the use of anæsthetic agents, in the year 1846, in dental surgery. The introduction of these agents added considerably to Mr. Robinson's reputation, and his practice became one of the most extensive in this country, and it so remained to the time of his death. Notwithstanding that the duties of his practice involved an immense amount of personal labor (his regular professional hours were from nine to five or six), Mr. Robinson considered it incumbent on him to exert himself towards elevating the profession of which he was so active a member, by means of organized education and association. In 1842 he did his best to establish a Dental Society; but the wretched jealousies of the many, and the apathy of nearly all, rendered his exertions abortive. His contributions to the literature of the profession were very useful and numerous, most of them being published anonymously. His work, entitled "Robinson on the Teeth," is well known, and has an extensive circulation. Amongst his articles furnished to American and other periodicals, may be mentioned that "On Filling the Teeth," contributed in 1845. "Observations upon the Importance and Value of a Knowledge of the Collateral Branches of Medicine and Surgery," contributed in 1847. "The History of Dental Surgery," contributed in 1845, etc. For some years prior to his death, he was engaged in compiling a more extensive work on Dental Surgery and Mechanics—this will probably appear, ere long, as we understand it is nearly completed.

On the 13th of July, 1849, Mr. Robinson received the appointment of Surgeon-Dentist to H. R. H., Prince Albert.

The attainments and position of Mr. Robinson attracted the attention of the authorities of the Baltimore College of Dental Surgeons, who, in the year 1846, on March 8th, conferred upon him the honorary degree of Doctor of Dental Surgery.

Such is a brief review of Mr. Robinson's life, with the exception of the leading part taken by him in the Great Dental Reformatory Movement, publicly commenced in 1856. It is not now necessary to recapitulate particulars which are amply recorded in the pages of this journal, and in those of its predecessor, the "Quarterly Journal of Dental Science;" suffice it to say, he was unanimously elected first President of the College of Dentists, in November, 1856. This post he filled with ability and success until the unfortunate differences which sprung up in 1858, on the Amalgamation Question. On this question he no doubt acted, as he thought, for the best, but his judgment was probably more at fault here than on any other occasion. The terms of amalgamation having been rejected by the majority at a special meeting of the members, Mr. Robinson resigned the Presidency. We have very good reason to believe that he looked back on this period with deep regret. When time had disclosed to him the fact, that the supporters of the College of Dentists (in its own *name*, according to the distinct arrangement of the original delegates from the College and frőm the Odontological Society), were right, and that he had failed to perceive an influence over his own free will. Thus, in 1860, on a vacancy occurring in the Treasurership, it was offered to him, and he readily accepted it. From this time his interest in the cause of independent action, as represented by the College, was warmly evinced, and every possible reparation towards counteracting the effects of any injury sustained by the College from his resignation of the Presidency.

During the past year, Mr. Robinson exerted all his energies in founding the National Dental Hospital. In this he was successful, and shortly before his death witnessed the opening of the Hospital in Great Portland street, and received the acknowledgments of its supporters, according to his due, as *the founder*. We believe the last meeting of any kind attended by him was that of the Committee of the Hospital, on the Thursday evening before his decease.

In character, as before observed, Mr. Robinson was most energetic. He was also of a kindly social disposition, and many are those in the profession who can testify to his cheerful and liberal hospitality. The loss of Mr. Robinson is a great one to the profession. Those with whom he was publicly associated paid him a last tribute of respect by officially attending his funeral at Highgate Cemetery, on the 11th March ult. The melancholy occasion witnessed the Council of the College of Dentists, and the Committee of the National Dental Hospital, meeting in this melancholy ceremony with his numerous other professional and private friends. Of those present, none, we feel convinced, will forget the solemnity attending the funeral.

---

We speak daily of the shortness and uncertainty of life, but we live on as if riches, and honors, and long years were before us. Only when death suddenly takes away some near relation or intimate friend, do we realize the truth of the various symbols by which the preacher and the sage have represented the insecurity of man's existence. Little did we think, when writing the article of the previous month advocating the cause of Dental Hospitals, that we should so soon be called upon to record the loss of one of the foremost promoters of these institutions.

Death has unexpectedly deprived the dental profession of one of its most noted members. The late Mr. James Robinson, whose loss is deplored not only by his relatives and friends, but by a large portion of the public, ranked amongst the honorable band of self-made men. He belonged to a class who not only labor with their hands, but whose brains create work for their hands to do. Always busily employed, however much such men accomplish, they are constantly seeking after something greater, and no sooner is one object fulfilled than new projects take the place of those which previously engaged their attention. Let us look back some quarter of a century, and the name of Mr. Robinson is as little known as that of any other man quietly pursuing the useful but unostentatious calling of a general chemist. Pass onwards to the end of that time when the fatal accident occurs which deprives him of existence. In America and Germany, his reputation as a dentist is well known. In England, his acquaintance amongst the dental profession is, probably, greater than any man's. In the enjoyment of a lucrative and extensive practice, the relation in which he stands to his patients is almost universally that of a personal friend, and the influence he possesses over them is most remarkable. The daily papers record his death as that of a public character; the medical and dental periodicals each pay a tribute of respect to his memory, and a large concourse of mourners attend his body to the grave. The unknown man has made for himself a world-wide reputation, and when he passes away, he receives honors, which, at least, in this country, have never been previously accorded to any member of the dental profession.

One naturally inquires what were the mental and physical endowments of the individual, which enabled him to accomplish, in a comparatively short space of time, what so many fail to perform in a life-time. Great activity, great energy, and an entire devotion to the work in hand, were among the marked traits of Mr. Robinson's character. To these must be added a large amount of self-confidence and self-esteem, qualities, which, when combined with the former, will do much to advance a man in life, and will enable him to succeed where the more cautious and timid are apt to pause, and to permit the golden opportunity to escape them. But while these qualities may be sufficient to ensure success, others of a more kindly and genial nature are requisite to produce that general popularity which Mr. Robinson enjoyed in an eminent degree. His extensive practice was necessarily accompanied by a large share of worldly prosperity; at the same time money was not the object of his ambition, and there are few who possessed a more liberal or hospitable spirit. His chief desire was to stand at the head of his profession, and to be the center from whence all progress and reform should appear to emanate. He has, indeed, a just claim to be placed among the first promoters of dental reform, seeing that he endeavored to bring about an organization of the profession some years before the foundation of the College of Dentists. The man who had fought his way by his own perseverance was not likely to be the one who would desire his profession to be in subjection to any other body, whatever might be the standing or position of the latter. Hence, Mr. Robinson was, from the first, a strenuous supporter of the College of Dentists, and remained so at the period of his death. The last work in which he was engaged was in the establishment of the National Dental Hospital. On no occasion were the peculiar energy and perseverance which characterized the man more strikingly displayed, or attended with more triumphant success. Men may arise who shall be more learned and equally skillful as practitioners, but it will be long before the dental profession will produce an individual who shall hold the same prominent and peculiar position as that in which Mr. Robinson stood, alike to the profession and to the general public. To the younger members of all professions and of all callings, he presents a striking example of what may be accomplished by a man of average talents, but possessed of a determined will, accompanied by a large amount of industry and perseverance.—*Dental Review, London.*

A great one has fallen. By his works he was known. He left a void that can not be filled. He left a noble example; let all emulate it. T.

